# Reducing minimum direct care staffing requirements in Florida nursing homes: implications and future directions

**DOI:** 10.1093/geroni/igaf122.3037

**Published:** 2025-12-31

**Authors:** Lindsay Peterson, Dylan Jester

**Affiliations:** University of South Florida, Tampa, Florida, United States; VA Palo Alto Healthcare System, Palo Alto, California, United States

## Abstract

A robust body of research associates higher numbers of direct care staff hours with better quality of care in nursing homes. More RN hours are associated with fewer regulatory deficiencies, pressure ulcers, urinary tract infections, and emergency department visits. More CNA hours are associated with fewer deficiencies, hospitalizations, and pressure ulcers. In 2022, the state of Florida passed legislation to reduce the minimum nursing care requirements for the roughly 700 nursing homes statewide. Prior to the reduction, Florida was a national model with a required 3.6 hours of direct care per resident per day, including 2.5 hours of Certified Nursing Assistant (CNA) care and 1 hour from a licensed nurse. The 2022 legislation reduced the CNA requirement to 2 hours of care per resident per day. Licensed nurses remained at 1 hour and other staff, such as feeding assistants, were authorized to fulfill the remaining 0.6 hours. This study’s purpose was to assess the effect of the change using staffing and quality measures data from the Centers for Medicare and Medicaid Services. We found that as of April 2021, Florida nursing home residents received, on average, 3.93 hours of direct nursing care per day. By October 2022, the number had dropped to 3.60. CNA time dropped the most, from 2.55 hours to 2.33 hours per resident per day. Additionally, we found during that same time an increase in resident emergency department visits and hospitalizations. We discuss the implications and nursing home staffing trends across the United States.

